# Giant edge state splitting at atomically precise graphene zigzag edges

**DOI:** 10.1038/ncomms11507

**Published:** 2016-05-16

**Authors:** Shiyong Wang, Leopold Talirz, Carlo A. Pignedoli, Xinliang Feng, Klaus Müllen, Roman Fasel, Pascal Ruffieux

**Affiliations:** 1 Nanotech@surfaces Laboratory, Empa, Swiss Federal Laboratories for Materials Science and Technology, 8600 Dübendorf, Switzerland; 2 NCCR MARVEL, Empa, Swiss Federal Laboratories for Materials Science and Technology, 8600 Dübendorf, Switzerland; 3 Department of Synthetic Chemistry, Max Planck Institute for Polymer Research, 55124 Mainz, Germany; 4 Department of Chemistry and Biochemistry, University of Bern, 3012 Bern, Switzerland

## Abstract

Zigzag edges of graphene nanostructures host localized electronic states that are predicted to be spin-polarized. However, these edge states are highly susceptible to edge roughness and interaction with a supporting substrate, complicating the study of their intrinsic electronic and magnetic structure. Here, we focus on atomically precise graphene nanoribbons whose two short zigzag edges host exactly one localized electron each. Using the tip of a scanning tunnelling microscope, the graphene nanoribbons are transferred from the metallic growth substrate onto insulating islands of NaCl in order to decouple their electronic structure from the metal. The absence of charge transfer and hybridization with the substrate is confirmed by scanning tunnelling spectroscopy, which reveals a pair of occupied/unoccupied edge states. Their large energy splitting of 1.9 eV is in accordance with *ab initio* many-body perturbation theory calculations and reflects the dominant role of electron–electron interactions in these localized states.

## Abstract (web summary)

The zigzag edges of graphene host edge-localized electronic states with aligned electron spins, but these states strongly interact with metallic substrates. Here, the authors measure the electronic structure of graphene nanoribbons after transferring them to an insulating support.

## Graphical abstract

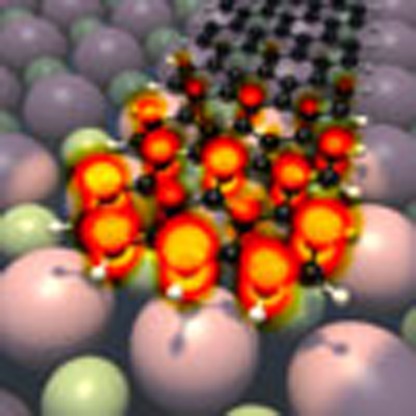

Recent advances in the fabrication of precise graphene nanostructures open the door to tailoring their electronic properties to the needs of specific applications. In the case of graphene nanoribbons (GNRs) with armchair edges, a bottom-up approach has been shown to deliver control over width and edge termination down to the atomic level^1^. This allows for precise tuning of the electronic band gap^2^,^3^,^4^ and optical response^5^ by adjusting the shape and coupling motifs of the molecular building blocks. Even more intriguing are graphene nanostructures with zigzag edges, which are predicted to host spin-polarized edge states by different levels of theory^6^,^7^,^8^,^9^,^10^. Although a significant number of theoretical studies have investigated specific graphene nanostructures with zigzag edges, predicting spin filtering properties^11^, half-metallic behaviour^12^ and spin confinement^13^, experimental results are scarce and widely affected by limited structural precision and/or pronounced interaction with the substrate. Previous experimental studies of graphene zigzag edges have concentrated mainly on metal-adsorbed graphene nanostructures, where the low-energy edge states may interact with the nearby electron reservoir. For graphene nanoislands on Ir(111), edge states are found to be completely suppressed^14^. On less reactive surfaces, such as Au(111)^15^,^16^,^17^,^18^,^19^,^20^, edge states have been observed for a variety of graphene nanostructures, even at interfaces between graphene and hexagonal boron-nitride^18^,^20^. However, the reported spectroscopic features of edge states vary greatly. For example, the energy splitting between occupied and empty edge states ranges from 0 eV (ref. 17) to 0.3 eV (ref. 19). These values are much smaller than expected from electronic structure calculations for structurally perfect zigzag edges within many-body perturbation theory, which predicts a splitting of ≈1.9 eV for the most strongly localized edge state^8^. Indeed, a recent study of the edges of graphene grown on silicon carbide^21^ reports a substantial energy splitting of up to 1.2 eV, however, the edges obtained from nanoparticle-assisted etching lack atomic precision. In summary, reducing both edge roughness and substrate interaction can be considered a prerequisite for studying the intrinsic electronic and magnetic structure of graphene zigzag edges.

Here, we focus on the electronic properties of the atomically precise zigzag edges formed at the termini of bottom-up fabricated armchair graphene nanoribbons (AGNRs). To decouple their electronic structure from the metal substrate, on which they are grown, we transfer the AGNRs onto NaCl islands by a scanning tunnelling microscopy (STM)-based multistep manipulation routine. Using scanning tunnelling spectroscopy (STS), we find that electronic decoupling of the edge states establishes a large energy splitting between occupied and unoccupied edge states. This is in accordance with *ab initio* many-body perturbation theory calculations, which we use to systematically distinguish between edge states localized at the zigzag edges and the energetically and spatially distinct states associated with the armchair edges in the GNRs under study.

## Results

### Short graphene zigzag edge on a thin insulator

**Figure 1:**
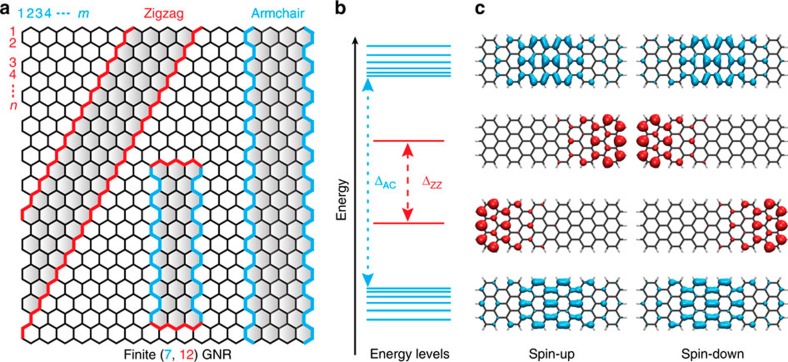
Electronic structure of finite graphene nanoribbons. (**a**) Cutting graphene into nanoribbons with different edge topologies. Indices (*m*, *n*) are used to denote the dimensions of a graphene nanoribbon (GNR) along the zigzag (*m*) and armchair direction (*n*), respectively. (**b**) Sketch of energy levels for a finite (7, 12) GNR, with Δ_AC_ and Δ_ZZ_ indicating the bulk band gap and the splitting of the localized states at the zigzag edges, respectively. (**c**) Kohn–Sham spin-orbitals of edge-localized states and energetically closest bulk states. Electrons with different spins are localized at opposing zigzag edges.

We focus on short AGNRs of width *m*=7, which are synthesized with atomic-scale precision on a Au(111) single crystal surface using a recently established bottom-up method^1^. The two armchair and two zigzag edges of the GNRs are atomically precise, monohydrogenated edges, as demonstrated by previous combined STM and atomic force microscopy studies^17^. Following Fig. 1a, the GNRs are denoted as (7, *n*) GNRs, where *n* specifies their length along the armchair direction in units of carbon zigzag lines. Finite (7, *n*) GNRs host two qualitatively different sets of electronic π-states. One set derives from the Bloch states of the bulk GNR, which are delocalized along the GNR. The short zigzag edges at the termini of the GNRs give rise to another set of states that are localized near the termini^10^. As sketched in Fig. 1b,c, these edge states (Tamm states^22^) are energetically isolated from the delocalized bulk states of the GNR, thus offering an experimental advantage over graphene nanostructures with long zigzag edges, where the energies of edge-localized and delocalized states are predicted to overlap^6^,^8^. Previous STS investigations of (7, *n*) GNRs on Au(111) indicated only one, possibly degenerate, edge state near the Fermi level, which may be explained by hole-doping of the GNR^17^. In order to characterize their intrinsic electronic structure, the GNRs thus need to be transferred to a different substrate—a process that is also required for future GNR-based applications^23^.

**Figure 2:**
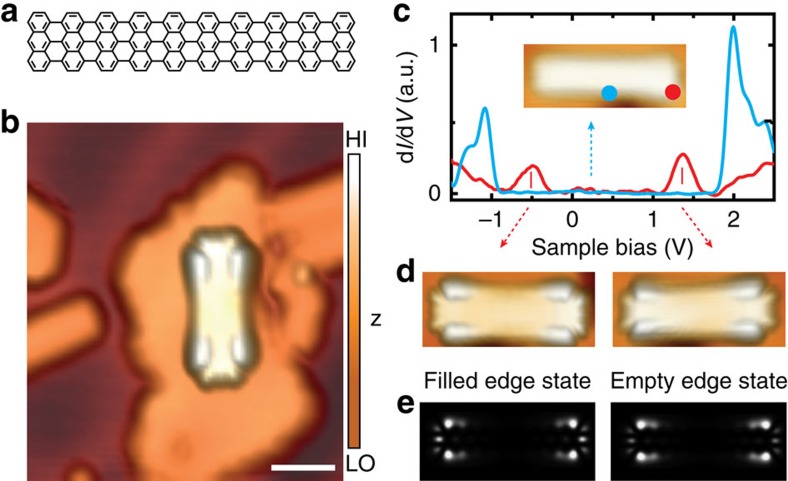
Electronic structure of (7, 20) graphene nanoribbon on NaCl monolayer. (**a**) Structural model of a (7, 20) GNR. Scale bar, 2 nm. (**b**) STM topography image of a (7, 20) GNR transferred onto a NaCl monolayer island through STM manipulation (*U*=−1.0 V, *I*=30 pA). Colour bar: HI=high; LO=low. (**c**) Differential conductance spectra measured in the centre (blue) and at a zigzag end (red) of the decoupled (7, 20) GNR. Inset: STM topography image at sample bias in the band gap of the ribbon (*U*=−0.5 V, *I*=30 pA). (**d**) STM topography images showing the orbital shapes of the occupied edge state (left, *U*=−1.0 V, *I*=30 pA) and the unoccupied edge state (right, *U*=1.4 V, *I*=30 pA). (**e**) Local density of states of corresponding Kohn–Sham orbitals at 4 Å distance above the GNR.

As the synthesis relies on the catalytic activity of the metal surface, the transfer onto an insulating substrate needs to occur *after* the synthesis. Here, we use atomically thin insulating NaCl films that are deposited directly onto the metal surface. In contrast to bulk insulators, these films still allow the electronic properties of adsorbates to be investigated by STM/STS, while considerably reducing their interaction with the metal substrate^24^. Through a novel four-step STM manipulation routine (see the Methods, Supplementary Fig. 1 and Supplementary Note 1), we transfer ribbons with lengths ranging from 2 to 10 nm onto a monolayer of NaCl without introducing any defects. The transfer process relies on the weak adhesion of defect-free (7, *n*) GNRs to the Au(111) growth substrate, which enables lateral manipulation and controlled pick-up of individual GNRs by the STM tip^22^. Figure 2b shows a typical STM scan of a (7, 20) GNR on NaCl. When positioning the STM tip above a zigzag end of the decoupled GNR, the differential conductance (d*I/*d*V*) spectrum exhibits two peaks centred at −0.5 and 1.3 V, as shown in Fig. 2c (see Supplementary Fig. 4 for a scan along the zigzag edge and Supplementary Fig. 5 for comparison with a reference spectrum on NaCl). Both peaks are well separated from the Fermi energy, thus excluding the possibility of (partial) charge transfer. STM images taken at these bias voltages clearly associate the peaks with electronic states localized at the zigzag-terminated ends. As shown in Fig. 2d, the shapes of filled and empty edge states are essentially identical. Their characteristic features, such as the broadening towards the very end of the GNR as well as the protrusions at the outermost carbon atoms, are in excellent agreement with the orbital densities of the corresponding states in Kohn–Sham density functional theory (DFT) calculations for freestanding GNRs, when the finite tip-sample distance is taken into account^25^ (cf. Fig. 2e).

### Theoretical treatment of edge state splitting

Being a theory of the electronic ground state, Kohn–Sham DFT is not designed to describe the charged excitations that take place in STS, which involve the addition/removal of electrons to/from the sample. Although the orbitals of the non-interacting Kohn–Sham system are often found to be accurate approximations of the corresponding quasiparticle wave functions^26^, the Kohn–Sham orbital energies are known to deviate significantly from quasiparticle excitation energies in many bulk insulators and molecules. In particular, the Kohn–Sham gap of standard semi-local DFT functionals (and even of the exact functional^27^) can severely underestimate the fundamental gap, defined as the difference between the ionization potential and the electron affinity. An accurate description of the fundamental gap needs to properly account for the interaction of the additional charge with the remaining electrons. This many-body effect of dynamical screening is captured naturally by many-body perturbation theory in the *GW* approximation. The framework provides accurate fundamental gaps, both for bulk insulators^28^ and molecules^29^, and has been applied successfully to GNRs of infinite length^8^.

**Figure 3:**
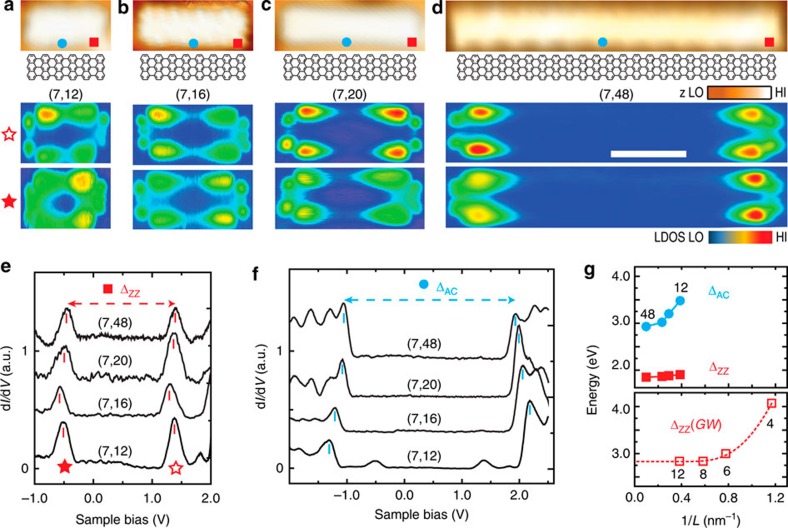
Length-dependent electronic structure of decoupled (7, *n*) graphene nanoribbons. (**a**–**d**) STM topography images and STS maps of empty and filled edge states of decoupled (7, 12), (7, 16), (7, 20) and (7, 48) GNRs on NaCl monolayer islands (*U*=−0.1 V, *I*=30 pA). Colour bars: LO=low, HI=high, LDOS=local density of states. (**e**,**f**) Differential conductance spectra taken at the terminus (**e**) and at the centre (**f**) of each ribbon shown in **a**–**d**. Scale bar, 2 nm. (**g**) Bulk band gap Δ_AC_, edge state splitting Δ_ZZ_ and calculated *GW* splitting Δ_ZZ_(*GW*) as a function of inverse GNR length (the dashed line serves as a guide to the eye).

Here we perform *ab initio* *GW* calculations for finite (7, *n*) GNRs, which can be viewed as open-shell molecules with one, singly occupied state localized at each terminus. Their fundamental gap is given by the energy splitting Δ_ZZ_ between the occupied and empty edge-localized states. A significant Kohn–Sham gap opens only in the spin-unrestricted formalism, where breaking of spin symmetry gives rise to staggered sublattice potentials^7^. Using the semi-local Perdew-Burke-Ernzerhof (PBE) functional^30^, a Kohn–Sham gap of 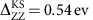 is obtained for lengths *n*⩾12. Starting from the PBE orbitals and orbital energies, we compute quasiparticle corrections in the *G*_0_*W*_0_ approximation. The fundamental gap Δ_ZZ_ is found to converge rapidly as a function of length, yielding a value of 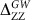
=2.8±0.1 eV for *n*⩾12 (see Fig. 3g for details), exceeding the Kohn–Sham gap by more than a factor of five. Note also that 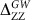 is larger than the *G*_0_*W*_0_ gap of maximum 1.9 eV between states localized at *extended* zigzag edges^8^, as expected from the additional confinement along the zigzag direction (see Supplementary Fig. 2 and Supplementary Note 2 for the link between the edge state of the (7, *n*) GNR and the edge states of extended zigzag edges). Direct comparison to experiment would require the inclusion of dynamical screening not only by the electrons of the GNR itself, but also by those of the NaCl monolayer and the underlying Au substrate, which is expected to lead to significant reduction of the fundamental gap^2^. Owing to computational constraints, we do not describe screening by the substrate quantitatively here (see refs 2, 31 for studies modelling such effects), but point out that the experimentally observed gap of Δ_ZZ_=1.9 eV is fully compatible with a fundamental gap of 2.8 eV, reduced by screening from substrate electrons.

### Separation between zigzag edges

To investigate finite size effects, ribbons of different lengths have been moved onto NaCl islands and inspected. Figure 3a–d shows STM topographies (upper panel) and STS maps (lower panel) of filled and empty edge states of (7, 12), (7, 16), (7, 20) and (7, 48) GNRs, respectively. In accordance with measurements on Au(111) as well as theory^17^,^32^, the edge states are found to be localized near the zigzag termini with a typical extent of 1.5 nm (see Supplementary Note 2 for a tight-binding analysis). Over the length range of 3–10 nm investigated here, Δ_ZZ_ is essentially independent of the separation between the zigzag edges, in accordance with the *G*_0_*W*_0_ predictions (cf. Fig. 3e and Supplementary Table 1). Similar observations have also been made on long, chemically etched zigzag GNRs on SiC, where a constant gap of 0.12 eV is reported for ribbons wider than 3 nm (ref. 21). In the case of long zigzag GNRs, however, the edge-localized states overlap energetically with delocalized states^8^, making it difficult to distinguish between the two in STS. For (7, *n*) GNRs, the additional quantum confinement at the short zigzag edges selects one particular wavelength along the zigzag edge and the corresponding edge state is energetically isolated from the delocalized states of (7, ∞) GNRs.

### Electronic band structure

We now turn to the delocalized bulk electronic states of decoupled (7, *n*) GNRs. Their width of 7=3 × 2+1 carbon dimer rows identifies (7, *n*) GNRs as members of the 3*m*+1-family of AGNRs, which have the largest band gaps^7^,^8^. Figure 3f shows d*I/*d*V* spectra taken at the centre of a (7, 12) GNR, featuring two sharp peaks at –1.2 and 2.3 V. These peaks indicate the onsets of the highest occupied and the lowest unoccupied bulk states, respectively, yielding a bulk band gap of Δ_AC_=3.5 eV. In contrast to the energy splitting Δ_ZZ_ of the localized edge states, we find that the bulk band gap Δ_AC_ decreases continuously from 3.5 eV for the shortest (7, 12) GNR to 2.9 eV for the longest (7, 48) GNR under study, as shown in Fig. 3g. This trend is rationalized by the decreasing longitudinal confinement of the associated bulk states that extend throughout the GNR. Figure 3g suggests that the value of 2.9 eV, measured for a GNR of 10 nm length, is converged within experimental accuracy, in agreement with length-dependent band gap studies of (7,*n*) GNRs on Au(111)^25^. Many-body perturbation theory calculations in the *GW* approximation predict a band gap of 3.7±0.1 eV for freestanding (7, ∞) GNRs^2^,^8^. Although the observed band gap of Δ_AC_=2.9 eV is still below this value, it is significantly larger than the 2.4 eV measured for the (7, ∞) GNR on Au(111)^25^, thus indicating considerably reduced screening by substrate electrons.

**Figure 4:**
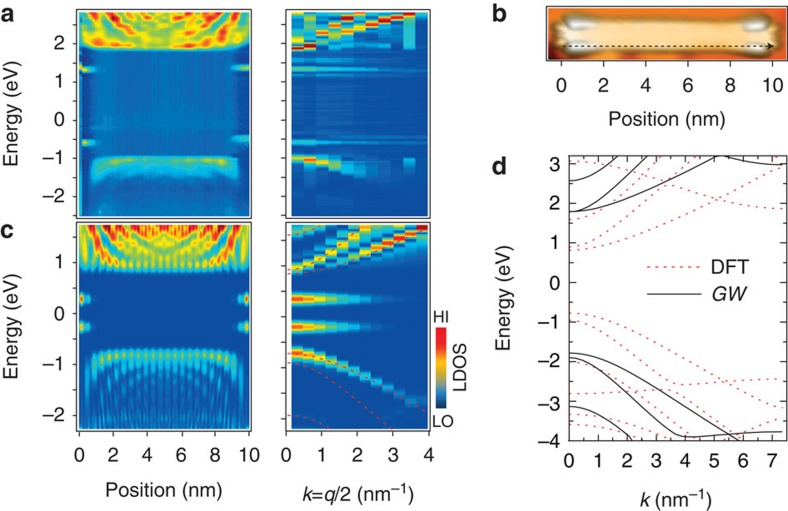
Band structure of decoupled (7, 48) graphene nanoribbon. (**a**) Left panel: Grid of d*I/*d*V* spectra (spaced 0.15 nm) taken along the armchair edge of a decoupled (7, 48) GNR. Right panel: Fourier transformed map revealing one occupied band and two unoccupied bands near the Fermi level. (**b**) STM topography of a decoupled (7, 48) GNR (*U*=−1 V, *I*=30 pA). (**c**) Left panel: DFT-based local density of states (LDOS) of (7, 48) GNR at 4 Å tip-sample distance (integrated across the ribbon). Right panel: Fourier transformed LDOS with DFT bands of infinite ribbons superposed as dashed red lines. Colour bar: LO=low, HI=high. (**d**) DFT and *GW* band structure of (7, ∞) GNR, aligned at the centre of the gap (zero energy).

Furthermore, the dispersion of the electronic states of a decoupled (7, 48)-GNR has been determined via Fourier transformed (FT) STS^25^. Figure 4a shows a grid of STS spectra taken along one armchair edge. In the colour map, both the edge states and the bulk states can be resolved. The bulk states show standing waves arising from scattering at the termini of the GNRs, in good agreement with the corresponding DFT-based FT-STS simulation of a (7, 48)-GNR (Fig. 4b). For example, at −1.4 V bias, four nodes are observed along both armchair edges. With decreasing bias, we observe three nodes, two nodes and one node at −1.2, −1.1 and −1.0 V, respectively (see Supplementary Fig. 3 for constant-current STS maps). To quantitatively investigate the electronic band dispersion, we perform a discrete FT to reciprocal space as shown in Fig. 4b. One occupied band and two unoccupied bands can be resolved, with effective masses of 0.32±0.04, 0.35±0.10 and 0.20±0.05 *m*_e_, respectively (*m*_e_ is the free electron mass). We note that the bands appear with different intensity in STS because of the finite tip-sample distance and refer interested readers to the corresponding study on Au(111) for details^25^. Although the effective masses are slightly smaller than those determined for the (7, ∞) GNRs directly on Au(111)^25^, the respective error bars overlap, indicating that the effective masses are largely unaffected by the electronic decoupling, despite the accompanying significant increase of the band gap. Figure 4c shows the DFT band structure of the (7, ∞) GNR and the corresponding many-body corrections within the *G*_0_*W*_0_ approximation. Although the band gap is found to open from 1.6 to 3.6 eV (ref. 8), the effective masses are found to decrease only by ∼10%. This finding is consistent with earlier work on graphene where many-body corrections to the local-density approximation give rise to a similar increase of the dispersion near the Fermi energy^33^.

## Conclusions

We have demonstrated the transfer of atomically precise (7, *n*) graphene nanoribbons from Au(111) to an insulating monolayer of NaCl without introducing any defects. The delocalized electronic states of (7, *n*) GNRs are separated energetically from the states localized at the zigzag termini, making it possible to investigate the effect of electronic decoupling on both classes of states separately. Using STS, the band gap between delocalized states is found to increase from 2.4 to 2.9 eV upon electronic decoupling, whereas no significant modification of the effective masses is observed. Furthermore, we find an energetic splitting of the states localized at the zigzag termini that has long been predicted for freestanding zigzag edges, but is missing completely in (7, *n*) GNRs on Au(111). Both its substantial size of Δ_ZZ_=1.9 eV and its independence on edge separation down to 3 nm are in agreement with *ab initio* many-body perturbation theory calculations of the GNRs' intrinsic electronic structure, indicating that decoupling by a single layer of NaCl indeed allows to study the intrinsic properties of graphene zigzag edges. We therefore expect that the experimental strategy established here will particularly benefit the eagerly awaited exploration of the low-energy spin physics at graphene zigzag edges using spin-sensitive methods.

## Methods

### Sample preparation and transfer procedure

Sample preparation and STM measurements were performed in an ultrahigh vacuum system (base pressure 1 × 10^−10^ mbar) using an Omicron low-temperature STM. The Au(111) substrate was cleaned by standard argon sputtering and annealing cycles. The GNRs were grown on Au(111) following the recipe by Cai *et al*.^1^. The sample temperature for the cyclodehydrogenation step was chosen such as to yield monohydrogenated termini^32^. NaCl powder was thermally evaporated at the sample held at room temperature. Immediately after NaCl deposition, the sample was transferred to the STM chamber and cooled down to 5 K for characterization. This results mostly in NaCl monolayers, as judged by their apparent height of 2.2 Å. To transfer a GNR onto NaCl, the GNR is picked up at one end by the STM tip^22^. Together with the GNR, the tip is then moved laterally above the NaCl monolayer, whereas the other end of the GNR still remains physisorbed on Au(111). After applying a voltage pulse of 3.0 V to release the ribbon, the tip is used to push the GNR fully onto the NaCl monolayer (see also Supplementary Fig. 1 and Supplementary Note 1). Transfer of GNRs onto bilayer NaCl was found to be more challenging, as partial adsorption of GNRs on NaCl bilayers was unstable. All d*I/*d*V* spectra were recorded using the lock-in technique with *U*_rms_=20 mV.

### Computational methods

Electronic structure calculations within the framework of density functional theory were performed with the PBE exchange-correlation functional^30^. Band structure calculations were carried out using the Quantum ESPRESSO package^34^. The electronic structure of the (7, 48) GNR was calculated with the CP2K code^35^, which expands the electronic wave functions on an atom-centred Gaussian-type basis set. After extrapolating the Kohn–Sham orbitals into the vacuum region^36^, STS simulations were performed in the Tersoff–Hamann approximation^37^ on a plane parallel to the planar GNR. Quasiparticle corrections were computed in the *G*_0_*W*_0_ approximation using the BerkeleyGW package^28^,^38^. The static dielectric matrix was calculated using a rectangular Coulomb-cutoff along the aperiodic directions^39^ and extended to finite frequencies via the generalized plasmon pole model^28^. In the calculation of the self-energy, the static remainder approach was used to speed up the convergence with respect to the number of empty bands^40^ (more details in Supplementary Note 3).

## Additional information

**How to cite this article:** Wang, S. *et al*. Giant edge state splitting at atomically precise graphene zigzag edges. *Nat. Commun.* 7:11507 doi: 10.1038/ncomms11507 (2016).

## Supplementary Material

Supplementary Information Supplementary Figures 1-5, Supplementary Table 1, Supplementary Notes 1-3 and Supplementary References
