# Supplementary material for: Giant edge state splitting at atomically precise graphene zigzag edges
Source: Nat Commun. 2016 May 16;7:11507. doi: 10.1038/ncomms11507 (PMC4873614; doi:10.1038/ncomms11507)
Supplement: Supplementary Information — Supplementary Figures 1-5, Supplementary Table 1, Supplementary Notes 1-3 and Supplementary References [file ncomms11507-s1.pdf]

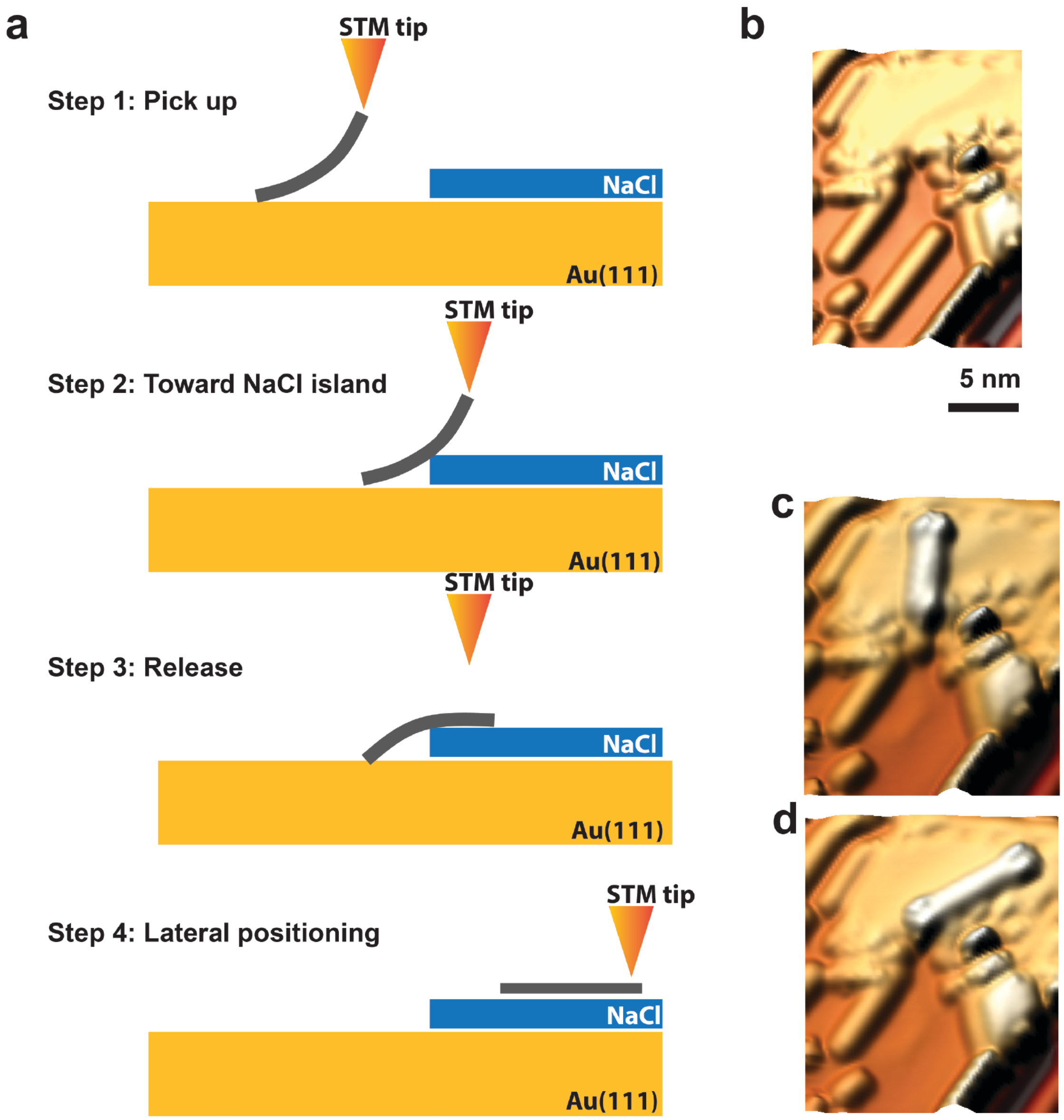

Supplementary Figure 1: STM manipulation routine for transfer of graphene nanoribbons onto NaCl monolayers. a, Schematic illustration of STM manipulation (cf. Supplementary Note 1). **b,** STM image (U = -0.1 V, I = 30 pA) of a (7, 48) GNR before manipulation. **c,** STM image (U = -0.1 V, I = 30 pA) after step 3, showing the ribbon partially adsorbed on NaCl. **d,** STM image (U = -0.1 V, I = 30 pA) after step 4, showing the ribbon fully adsorbed on the NaCl monolayer.

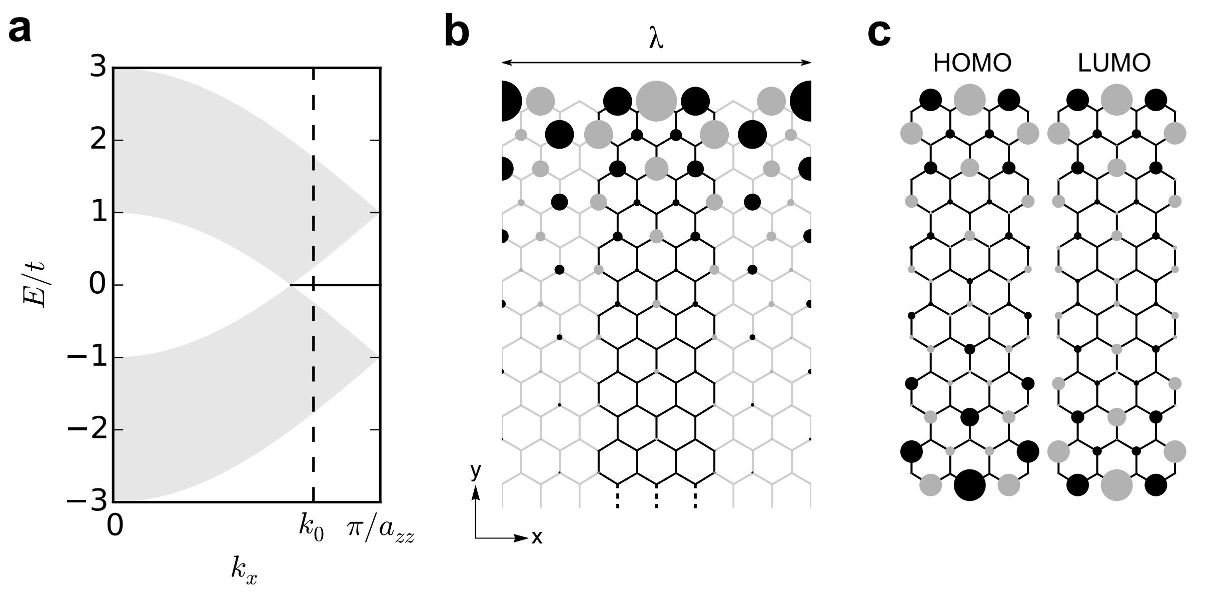

Supplementary Figure 2: Edge states at infinite and finite zigzag edges. a, Band structure of graphene projected onto the zigzag direction. The solid black line indicates the states localized at the zigzag edge of a semi-infinite graphene sheet. $k_0 = 0.75 \frac{\pi}{a_{zz}}$. **b,** Semi-infinite graphene sheet and linear combination of Bloch waves with wave-length $\lambda = 8a_{zz}$, satisfying the boundary conditions for (7, ∞) GNRs. The circle area is proportional to the electron density, while grey/black indicates the sign of the wave function. **c,** Highest occupied molecular orbital (HOMO, "bonding") and lowest unoccupied molecular orbital (LUMO, "antibonding") of (7,12) GNR.

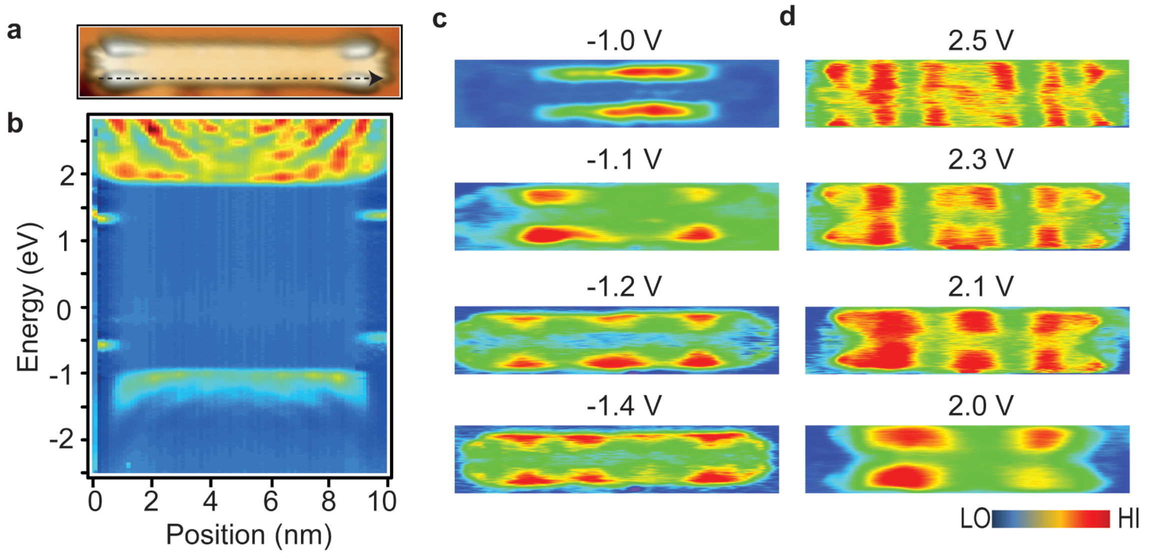

Supplementary Figure 3: Particle-in-a-box states of a (7, 48) graphene nanoribbon. a, STM image (U = -1 V, I = 30 pA) of a (7, 48) GNR. **b,** Grid of *dI/dV* spectra (spaced 0.15 nm) along the decoupled (7,48) GNR. **c,** constant-current *dI/dV* maps at different negative bias voltages exhibiting particle-in-a-box features due to quantum confinement. **d,** constant-current *dI/dV* maps at different positive bias voltages. The apparent localization of STS intensity near the armchair edges of the GNR arises due to the finite tip-sample distance, at which only the exponential tails of the GNR states are probed. It does *not* reflect edge-localization of the states close to the GNR atoms. The effect of finite

tip-sample distance on these states is well understood[1] and included in the STS simulations shown in Figures 2e and 4c.

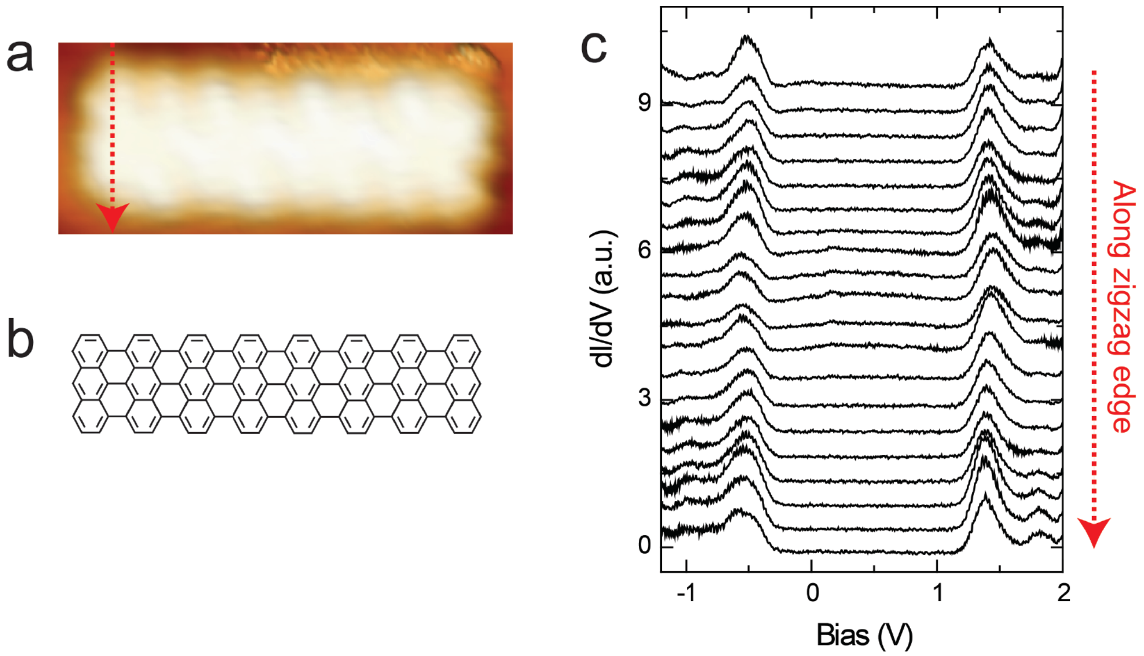

Supplementary Figure 4: Differential conductance spectra taken along zigzag edge. a, STM image (3.3 nm * 1.4 nm, *U* = -0.1 V, *I* = 30 pA) of a decoupled (7, 16) GNR. **b,** Structural model of the ribbon. **c,** *dI/dV* spectra taken along the zigzag edge, as indicated in **a**. All spectra show a pair of edge states with an energy splitting of 1.9 eV.

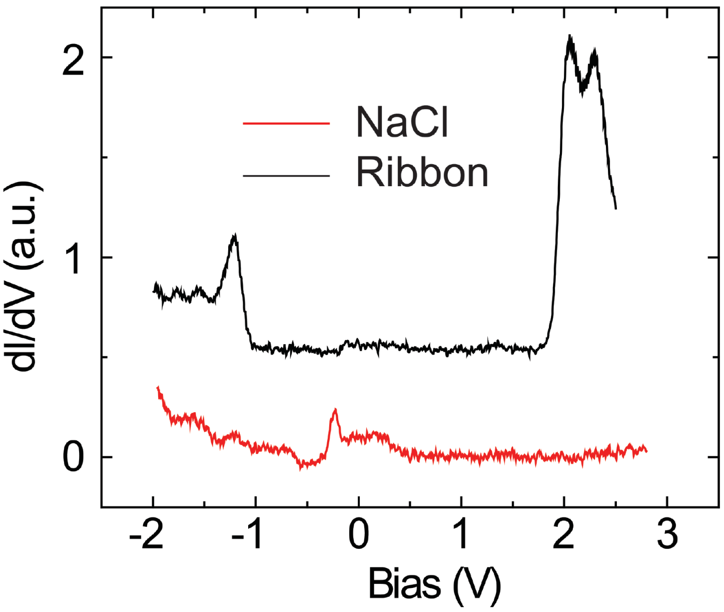

Supplementary Figure 5: Reference spectrum on NaCl. *dI/dV* spectrum taken on a NaCl island (red) shows Au(111)-derived interface state at *U* = –0.3 V. *dI/dV* spectrum taken on decoupled ribbon using the same tip (black) still shows the interface state, but with strongly reduced intensity.

	n=2	n=4	n=6	n=8	n=12
**Δ_{ZZ} (PBE) [eV]**	2.33	0.90	0.58	0.55	0.54
**Δ_{ZZ} (G_0W_0) [eV]**	6.49	4.03	3.00	2.86	2.85

Supplementary Table 1: Edge state splitting of finite (7,*n*) graphene nanoribbons. Comparing Δ_{ZZ} obtained from the PBE Kohn-Sham gap and from the G_0W_0 gap. GNR length is specified by the number of zigzag lines *n*, as defined in Figure 1a of the main text.

Supplementary Note 1: Transfer of GNRs onto NaCl monolayer islands

Individual (7,n) GNRs are physisorbed on Au(111), where they can be moved laterally by an STM tip. However, direct pushing of ribbons onto NaCl monolayer islands was found impossible. The apparent height of NaCl (2.2 Å) is higher than that of GNRs (1.8 Å) and, moreover, the NaCl islands themselves are physisorbed on Au(111) and easily moved by an STM tip. Recently, Koch *et al.* have demonstrated that individual ribbons can be picked up by an STM tip[2]. Inspired by their work, we have developed an STM manipulation routine (cf. Supplementary Figure 1) to transfer bottom-up fabricated GNRs from the metal substrate onto insulating NaCl monolayers in order to access their decoupled electronic structure. We have successfully achieved the transfer of GNRs with lengths ranging from 3 nm to 10 nm without introducing any defects. The STM manipulation routine consists of four steps: (1) pick-up of one end of a GNR by approaching and retracting the STM tip with low bias (≈-50 mV); (2) lateral movement of the tip (together with the GNR) above the NaCl island; (3) release of the ribbon by a voltage pulse of 3.0 V, leaving the GNR partially adsorbed on NaCl and partially on the metal surface; and (4) lateral positioning of the GNR fully onto the NaCl monolayer. Step 3 was introduced because releasing a GNR from the tip was found much easier when one end of the GNR is still adsorbed on Au(111). Transfer of GNRs onto bilayer NaCl has also been attempted, but was found to be much more challenging due to a lack of stable adsorption of the GNRs on NaCl bilayers in step 3.

Supplementary Note 2: Finite and infinite zigzag edges within tight binding

Single-orbital nearest-neighbor tight binding is a simple, yet instructive model that provides qualitative insights into graphene's π-electronic structure[3]. Within this model, the edge states of the (7,∞) GNR directly derive from those found at infinite zigzag edges, as is discussed in the following.

The corresponding Hamiltonian is given by

$$\mathcal{H} = -t \sum_{<ij>} c_j^\dagger c_i + c_i^\dagger c_j$$

where the sum extends over distinct pairs of nearest neighbors, $c_j^\dagger, c_i$ are the electron creation and annihilation operators on carbon sites i, j, and $t \approx 3$ eV is the hopping matrix element between nearest neighbors.

This model can be solved analytically both for graphene and for armchair and zigzag graphene nanoribbons[4]. Supplementary Figure 5a shows the resulting band structure of graphene, projected onto the zigzag direction. The solid black line indicates the non-bonding, singly occupied zero-energy states localized at the zigzag edge of a semi-infinite graphene sheet, as sketched in Figure S4b. The degree of localization of these states depends on the wave vector k along the zigzag direction. Perpendicular to the zigzag edge, the wave functions ψ_k decay exponentially with decay constant $\alpha_k = -2\ln\left(2\cos\left(\frac{ka_{zz}}{2}\right)\right)/a_{ac}$,[4] ranging from complete delocalization at $k = \frac{\pi}{a_{zz}}\frac{2}{3}$ $(\alpha_k = 0)$ to complete localization at $k = \frac{\pi}{a_{zz}}$ $(\alpha_k = \infty)$. Here, $a_{ac} = \sqrt{3}a_{zz} = 3a$, where $a \approx 0.142$ nm denotes the carbon-carbon bond length in graphene.

Supplementary Figure 5b views the (7, ∞) GNR as a slice of the semi-infinite graphene sheet, with the additional boundary condition that the wave function must vanish at the first carbon sites *outside* the GNR. For the (7, ∞) GNR these sites are separated by $\Delta x = (m+1)\frac{a_{zz}}{2} = 4a_{zz}$. This boundary condition is easily satisfied by an adequate superposition of $\psi_{k_0^+}$ and $\psi_{k_0^-}$ with crystal wave vectors $k_0^\pm = \frac{\pi}{a_{zz}} \pm \frac{2\pi}{\lambda} = \frac{\pi}{a_{zz}}\left(1 \pm \frac{1}{4}\right)$, as shown in panel 5b. Within tight binding, the orbital shape of the edge states of the (7, ∞) GNR thus directly derives from a superposition of two Bloch states of the semi-infinite graphene sheet. In particular, it shares their decay constant $\alpha_{k_0} \approx 1.3/\mathrm{nm}$ (with decay constant $2\alpha_{k_0} \approx 2.5/\mathrm{nm}$ for the charge density).

For finite (7,*n*) GNRs, the edge states of the two termini overlap. This gives rise to a finite energy splitting Δ_{zz} between a bonding and an anti-bonding linear combination of edge states. Supplementary Figure 5c depicts these states for the (7,12) GNR. In the length range $n \geq 12$ investigated experimentally, the weak overlap between states at the two termini gives rise to negligible splittings $\Delta_{ZZ} < 5$ meV.

Supplementary Note 3: Computational details

Electronic structure calculations were performed within the framework of density functional theory (DFT), using the PBE generalized-gradient approximation to the exchange-correlation functional as implemented in the Quantum ESPRESSO package[5]. The Kohn-Sham orbitals were expanded on a plane-wave basis set with energy cutoff at 150 Ry, using norm-conserving pseudopotentials.

For the finite (7,*n*) GNRs, the simulation cell was chosen at least twice as large as the 10^{-5} $/a_0^3$ isosurface of the charge density in order to enable the Coulomb-cutoff technique[6] in the GW calculations. For the (7, ∞) GNR, 18 Å of vacuum were introduced in the directions perpendicular to the GNR axis, while 16 k-points were used to sample the first Brillouin zone. The lattice parameter of the (7,∞) GNR was determined to be 4.285 Å and atomic positions were relaxed until the forces acting on the atoms were below 3 meV/Å.

Quasi-particle corrections were computed within the framework of many-body perturbation theory, using the G_0W_0 approximation to the self-energy as implemented in the BerkeleyGW package[7,8]. The electronic structure from DFT was recalculated using 60 Ry plane-wave cutoff (and 64 k-points in the first Brillouin zone for the (7, ∞) GNR). We have computed sufficient numbers of empty states to cover the energy range up to 2.1 Ry (infinite GNR) and 1.6 Ry (finite GNRs) above the highest occupied band. The static dielectric matrix ε was calculated in the random phase approximation with 8 Ry cutoff for the plane-wave basis. ε^{-1} was extended to the real frequency axis using the generalized plasmon-pole model by Hybertsen and Louie[7]. A rectangular Coulomb-cutoff was employed along the aperiodic dimensions as described in reference[6]. In the calculation of the self-energy, the static remainder approach was used to speed up the convergence with respect to the number of empty bands[9].

With regard to the magnetic structure, the PBE functional predicts ultrashort GNRs of lengths *n*=2 (anthracene) and *n*=4 (bisanthene) to have a spin-unpolarized ground state. From length *n*=6 onwards, PBE finds a ground state with spin-polarized, antiferromagnetically coupled edge states. The energy difference between the antiferromagnetic (spin-singlet) and the ferromagnetic (spin-triplet) solution, however, decays exponentially with GNR length, reaching ≈1 meV already for *n*=12, the shortest GNR length investigated experimentally. Similar findings have been obtained previously by the complete active space self-consistent field (CASSCF) method[10].

Supplementary References

1. Söde, H. *et al.* Electronic band dispersion of graphene nanoribbons via Fourier-transformed scanning tunneling spectroscopy. *Phys. Rev. B* **91,** 045429 (2015).
2. Koch, M., Ample, F., Joachim, C. & Grill, L. Voltage-dependent conductance of a single graphene nanoribbon. *Nat. Nano.* **7,** 713–717 (2012).
3. Saito, R., Dresselhaus, G. & Dresselhaus, M. S. Physical Properties of Carbon Nanotubes (Imperial College Press, London, 1998)
4. Katsunori, W., Ken-ichi, S., Takeshi, N. & Toshiaki, E. Electronic states of graphene nanoribbons and analytical solutions. *Sci. & Tech. Adv. Mater.* **11**, 054504 (2010).
5. Giannozzi, P. *et al.* QUANTUM ESPRESSO: a modular and open-source software project for quantum simulations of materials. *J. Phys.: Condens. Matter* **21**, 395502 (2009).
6. Rozzi, C. A., Varsano, D., Marini, A., Gross, E. K. U. & Rubio, A. Exact Coulomb cutoff technique for supercell calculations. *Phys. Rev. B* **73**, 205119 (2006).
7. Hybertsen, M. S. & Louie, S. G. Electron correlation in semiconductors and insulators: Band gaps and quasiparticle energies. *Phys. Rev. B* **34**, 5390-5413 (1986).
8. Deslippe, J. *et al.* BerkeleyGW: A massively parallel computer package for the calculation of the quasiparticle and optical properties of materials and nanostructures. *Comput. Phys. Commun.* **183**, 1269-1289, (2012).
9. Deslippe, J., Samsonidze, G., Jain, M., Cohen, M. L. & Louie, S. G. Coulomb-hole summations and energies for GW calculations with limited number of empty orbitals: A modified static remainder approach. *Phys. Rev. B* **87**, 165124 (2013).
10. Konishi, A., Hirao, Y., Kurata, H. & Kubo, T. Investigating the edge state of graphene nanoribbons by a chemical approach: Synthesis and magnetic properties of zigzag-edged nanographene molecules. Solid State Commun. **175-176**, 62–70 (2013).
